# Editorial: Generative adversarial networks in cardiovascular research

**DOI:** 10.3389/fcvm.2023.1307812

**Published:** 2023-10-23

**Authors:** Qiang Zhang, Tolga Cukur, Hayit Greenspan, Guang Yang

**Affiliations:** ^1^ Division of Cardiovascular Medicine, Radcliffe Department of Medicine, University of Oxford, Oxford, United Kingdom; ^2^ Big Data Institute, University of Oxford, Oxford, United Kingdom; ^3^ Department of Electrical and Electronics Engineering, Bilkent University, Ankara, Türkiye; ^4^ National Magnetic Resonance Research Center (UMRAM), Bilkent University, Ankara, Türkiye; ^5^ Department of Biomedical Engineering, Tel Aviv University, Tel Aviv, Israel; ^6^ Department of Radiology, Icahn School of Medicine, Mount Sinai, New York, NY, United States; ^7^ Bioengineering Department and Imperial-X, Imperial College London, London, United Kingdom; ^8^ National Heart and Lung Institute, Imperial College London, London, United Kingdom; ^9^ Cardiovascular Research Centre, Royal Brompton Hospital, London, United Kingdom; ^10^ School of Biomedical Engineering & Imaging Sciences, King’s College London, London, United Kingdom

**Keywords:** deep generative models, generative adversarial networks (GAN), echocardiography (Echo), cardiovascular magnetic resonance, segmentation (Image processing)

**Editorial on the Research Topic** Generative adversarial networks in cardiovascular research

Deep generative models are a family of neural networks capable of learning the data distribution from a large set of training samples and then generating realistic new data samples. They are among the most exciting technical breakthroughs in deep learning in recent years. A popular example is Generative adversarial networks (GAN) (1), which leverage a game-theoretic interplay between a generator and adversarial discriminator for an implicit characterization of the data distribution. In cardiovascular medicine, GANs are increasingly adopted in a wide range of applications for analysing cardiovascular MRI, echocardiography, electrocardiography and patient characteristics. This Research Topic has collected articles on the application of deep GAN models to left atrial appendage selection for surgical occlusion (Zhu et al.), function analysis in coronary artery stenosis (Yong et al.), late gadolinium enhancement scar assessment (Gonzales et al.), and strain analysis (Deng et al.), using echocardiography and cardiac MRI.

In Zhu et al., an adversarial-based latent space alignment framework has been proposed for left atrial appendage (LAA) segmentation in transesophageal echocardiography (TEE). LAA segmentation and quantification are crucial in guiding the surgical procedure for the treatment of LAA-associated ischaemic strokes. However, it is challenging on TEE due to TEE image artefacts, noise and highly variable LAA structure. To address this challenge, the authors encoded the prior knowledge of LAA shapes in a latent feature space and adopted generative adversarial learning to align the automated segmentation with the prior knowledge in the latent space, therefore constraining the segmentation results. The approach was validated on 1,783 TEE images and achieved superior performance with a Dice Similarity Coefficient (DSC) of 0.83. This work demonstrated the effectiveness of GANs in enhancing deep-learning models for challenging image modalities.

Yong et al. combined a GAN approach with intravascular ultrasound (IVUS) for non-invasive function analysis of coronary artery stenosis. The current clinical standard for coronary artery function evaluation is invasive fractional flow reserve (FFR). However, IVUS, a procedure routinely used for morphological assessment, has the potential to provide function evaluation simultaneously. To assess this approach, 92 patients who received both IVUS and FFR assessments were retrospectively identified. The authors employed a SegAN (2) to automatically segment the arterial lumen contours from IVUS images, which achieved a high DSC of 0.95 and 0.97 for lumen and media–adventitia border delineation. This allows accurate calculation of IVUS-FFR in good agreement with invasive FFR (*r* = 0.94), and a high diagnosis accuracy of 90.7%. Powered by deep generative models, IVUS demonstrated the feasibility of achieving comparable diagnostic performance with invasive FFR, with significantly lower computation time.

Gonzales et al. validated an approach of augmenting training samples with GAN to improve the accuracy of segmentation of cardiac MRI late gadolinium enhancement (LGE)—the imaging standard for myocardial scar assessment. The study leveraged GAN-generated virtual native enhancement (VNE) (3, 4)—a new, gadolinium-free modality that resembles LGE—to expand the training set. A dataset comprising 4,716 LGE images (from 1,363 patients with hypertrophic cardiomyopathy and myocardial infarction) was retrospectively collated. LGE data were augmented with a GAN-based generator to produce VNE images. The results demonstrated that incorporating GAN-generated VNE data into the training process consistently led to enhanced segmentation performance: the models trained on only LGE yielded a DSC of 0.835, 0.838 for LGE and VNE segmentation; whereas the models trained on both LGE and VNE yield higher DSC of 0.845, 0.845. Additionally, the individual segmentation performance of the model trained with only LGE data, including extensive data augmentation (5) (0.846) was also surpassed by the same framework when the VNE data were added (0.851). This work showed data augmentation using generative models as an effective approach to improving deep learning training, especially in the scenario of limited training data.

Additionally, in Deng et al., a deep learning approach has been developed for automated strain analysis on echocardiography. Strain analysis using echocardiography has great potential to offer rapid heart function assessment in routine clinical workflow. However, it requires myocardial segmentation, which is challenging in echocardiography. The authors developed a 3D U-Net and an optical-flow network to segment the LV myocardium, track the motion, and calculate the longitudinal strain. The AI-based echocardiography interpretation demonstrated a good agreement (Spearman correlation of 0.9) with the traditional semi-automatic speck tracking echocardiography (STE), with no significant bias (mean bias −1.2 ± 1.5%), whilst much faster (15 s vs. 5–10 min). Further development of generative models to learn the prior and distribution of a representative, real in-vivo data bank may help to translate this echocardiography technique into clinical practice.

In summary, these articles have highlighted the significant potential of deep generative models in revolutionising cardiac imaging, particularly in addressing intricate tasks and various image modalities. Looking ahead, we foresee a surge in upcoming research focused on fine-tuning and utilising deep generative models for a broader range of applications. These may involve reducing doses, rectifying missing modalities, augmenting data, refining image reconstruction (6), precise segmentation (7), accurate tracking of anatomical features, and dependable classification within the field of cardiovascular medicine.
